# Investigation of Thermoelectric Properties in Altermagnet RuO_2_

**DOI:** 10.3390/nano15141129

**Published:** 2025-07-21

**Authors:** Jun Liu, Chunmin Ning, Xiao Liu, Sicong Zhu, Shuling Wang

**Affiliations:** 1 Hubei Province Key Laboratory of Systems Science in Metallurgical Process, The State Key Laboratory for Refractories and Metallurgy, Collaborative Innovation Center for Advanced Steels, International Research Institute for Steel Technology, Wuhan University of Science and Technology, Wuhan 430081, China; jl@wust.edu.cn (J.L.); ncm12312312@wust.edu.cn (C.N.); 15342338918m@sina.com (X.L.); 2 School of Mathematics and Physics Science and Engineering, Hebei University of Engineering, Handan 056038, China

**Keywords:** TIMR, altermagnet, thermoelectric

## Abstract

An altermagnet, characterized by its distinctive magnetic properties, may hold potential applications in diverse fields such as magnetic materials, spintronics, data storage, and quantum computing. As a prototypical altermagnet, RuO_2_ exhibits spin polarization and demonstrates the advantageous characteristics of high electrical conductivity and low thermal conductivity. These exceptional properties endow it with considerable promise in the emerging field of thermal spintronics. We studied the electronic structure and thermoelectric properties of RuO_2_; the constructed RuO_2_/TiO_2_/RuO_2_ all-antiferromagnetic tunnel junction (AFMTJ) exhibited thermally induced magnetoresistance (TIMR), reaching a maximum TIMR of 1756% at a temperature gradient of 5 K. Compared with prior studies on RuO_2_-based antiferromagnetic tunnel junctions, the novelty of this work lies in the thermally induced magnetoresistance based on its superior thermoelectric properties. In parallel structures, the spin-down current dominates the transmission spectrum, whereas in antiparallel structures, the spin-up current governs the transmission spectrum, underscoring the spin-polarized thermal transport. In addition, thermoelectric efficiency emphasizes the potential of RuO_2_ to link antiferromagnetic robustness with ferromagnetic spin functionality. These findings promote the development of efficient spintronic devices and spin-based storage technology for waste heat recovery and emphasize the role of spin splitting in zero-magnetization systems.

## 1. Introduction

Spin caloritronics [1,2], a significant research direction in spintronics, investigates the interconnection between spin and thermal transport in materials, potentially enabling new thermoelectric conversion, waste heat recovery, and information processing technologies. The spin Seebeck effect (SSE), as one of the core topics in spin caloritronics, explores the thermally induced spin current and associated spin voltage in magnetic materials, offering a feasible mechanism to generate pure spin current [3,4,5,6]. Spin waves and conduction electrons are widely recognized as two primary carriers mediating spin-Seebeck currents [7]. Crucially, the efficiency of spin and thermal transport mediated by these carriers, particularly conduction electrons, is fundamentally governed by their scattering mechanisms. The electron scattering mechanism refers to the process in which electrons collide with other particles (such as other electrons, impurity atoms, lattice defects, etc.) while moving in a material, thereby changing their direction of motion and energy. In conductive materials, electrons are the main charge carriers, and they move under the influence of an electric field. However, due to the inhomogeneity within the material and the presence of other particles, the trajectory of electron motion is altered. Similarly, lattice vibrations play a key role. Phonons are the quantized representation of lattice vibrations. The phonon scattering mechanism, particularly relevant to electron–phonon interaction, refers to the process in which electrons interact with lattice vibrations (phonons) and change their state of motion. In solid materials, atoms vibrate slightly around their equilibrium positions, and these vibrations can propagate through the lattice. When electrons interact with these lattice vibrations, phonon scattering occurs. Nevertheless, the relationship between spin-dependent thermal transport and material symmetry persists as a critical challenge in developing high-efficiency spin-caloritronic devices [8]. Understanding these scattering mechanisms, their dependence on material structure, temperature, and magnetic order, is therefore paramount for unraveling the complexities of the SSE and addressing the symmetry-related challenge to achieve enhanced device performance.

Recently identified as a distinct category of antiferromagnetism, altermagnetism exhibits momentum-locked spin splitting and alternating spin polarizations in both real-space (crystalline structure) and momentum-space (band structure) [9,10]. This emergent phenomenon has garnered significant interest due to its exceptional properties [9,10,11,12,13,14,15,16]. Similarly to conventional antiferromagnetic (AFM) materials, altermagnets possess zero net magnetization; however, they manifest nonrelativistic spin splitting along high-symmetry band structure lines, enabling ferromagnetic (FM)-like behavior under specific conditions. This hybrid character establishes altermagnets as a link between AFM and FM systems, creating new avenues for spin manipulation in zero-magnetization environments [8]. Certain magnetic space groups (MSGs) with the broken *TPτ* and *Uτ* symmetries support this nonrelativistic spin splitting, where *T*, *P*, *U*, and *τ* denote the time reversal, spatial inversion, spinor symmetry, and the half lattice translation [12,13,14]. Given magnetism’s expansion through altermagnetism, integrating altermagnetic electrodes into magnetic tunnel junctions (MTJs) [17,18,19] represents a strategic progression toward achieving enhanced tunneling TMR effects and experimentally validating theoretical predictions.

MTJs comprising the FM/insulator barrier/FM sandwich structure [20,21,22] serve as the fundamental component of spintronic devices in applications such as nonvolatile magnetic random-access memories (MRAMs) and magnetic sensors [23,24]. By altering the magnetization orientation of the two FM electrodes between antiparallel and parallel configurations, an MTJ transitions between high- and low-resistance states—termed the TMR effect—which forms the operational basis of MTJ devices. Notwithstanding these advantages, conventional FM-based MTJs exhibit stray magnetic fields and constrained thermal stability, impeding their scalability and energy efficiency [25]. Conversely, AFM materials possess inherent benefits over FM materials: absence of stray magnetic fields, enhanced robustness against external fields, pronounced anisotropy, ultrafast spin dynamics, and appealing characteristics for AFM spintronics. Traditionally, however, the absence of global spin polarization prevented AFM materials from being utilized as MTJ electrodes. This longstanding perspective is now disrupted by altermagnetism, where momentum-dependent spin splitting facilitates localized spin currents without net magnetization [8].

Recent theoretical and experimental advances have confirmed that AFMTJs can produce the TMR effect when employing spin-splitting AFM electrodes, including RuO_2_ [16,26], Mn_3_Sn [27,28], and Mn_3_Pt [29]. Notably, RuO_2_ exhibits exceptional stability in acidic environments and tunable electronic structures, as evidenced by its recent applications in oxygen evolution reactions (OER) and solid-state catalysis [30,31], yet its potential in spin-dependent thermoelectric transport remains largely unexplored [32].

RuO_2_ demonstrates exceptional multifunctional properties, establishing it as a leading candidate for advanced spintronic and spin-caloritronic applications. Being a metallic altermagnet, RuO_2_ displays distinct spin-splitting in its electronic band structure, alongside high electrical conductivity [33] and ultralow thermal conductivity, making it an exceptional thermoelectric material. Research has confirmed the presence of a giant crystal Nernst effect and crystal thermal Hall effect within this material, which exhibit strong anisotropy concerning the Néel vector [34]. The significant crystal thermal transport stems mainly from three sources of Berry’s curvature in momentum space: the Weyl fermions due to crossings between well-separated bands, the strong spin-flip pseudo-nodal surfaces, and the weak spin-flip ladder transitions, defined by transitions among very weakly spin-split states of similar dispersion crossing the Fermi surface. Furthermore, it has been revealed that the anomalous thermal and electrical transport coefficients in RuO_2_ are linked by an extended Wiedemann–Franz law in a temperature range much wider than expected for conventional magnets. These findings suggest that altermagnets may play a leading role in realizing spin-caloritronic concepts unattainable with ferromagnets or antiferromagnets.

In this work, we systematically investigate the electronic structure, thermoelectric properties, and thermal spin transport characteristics of the altermagnet RuO_2_. Our results demonstrate that RuO_2_ exhibits distinct spin-splitting in its band structure, along with high electrical conductivity and low thermal conductivity. Through first-principle calculations, we further explore the spin current and thermal transport properties of RuO_2_/TiO_2_/RuO_2_ MTJs. Compared with prior studies on RuO_2_-based antiferromagnetic tunnel junctions, the novelty of this work lies in the thermally induced magnetoresistance based on its superior thermoelectric properties [26,35]. Our findings highlight the significant potential of altermagnetic RuO_2_ in applications related to thermal spintronics.

## 2. Theoretical Methods

First-principle calculations are performed based on the density functional theory (DFT) [36] as implemented in the Vienna ab initio simulation package (VASP) [37,38]. The pseudopotentials are described using the projector augmented wave (PAW) method [39], and the exchange-correlation functional is treated within the generalized gradient approximation (GGA) developed by Perdew, Burke, and Ernzerhof (PBE) [40]. The transport properties are computed using the non-equilibrium Green’s function formalism (DFT + NEGF approach) [41,42] and implemented in QuantumATK using its relaxed atomic structure. In QuantumATK, we used the nonrelativistic SG15 pseudopotentials [43], and k-point meshes of 17 × 17 × 25 for bulk RuO_2_ and TiO_2_ and 6 × 6 × 303 for RuO_2_/TiO_2_/RuO_2_ AFMTJ. The spin-polarized GGA + U [44,45] method with U_eff_ = 1.2 eV on Ru 4d [35] orbitals and U_eff_ = 5 eV on Ti 3d [35] orbitals is used in the calculations. These parameters have been well tested to ensure that the electronic structure around EF calculated by QuantumATK is consistent with that calculated by VASP.

## 3. Results and Discussion

**Figure 1:**
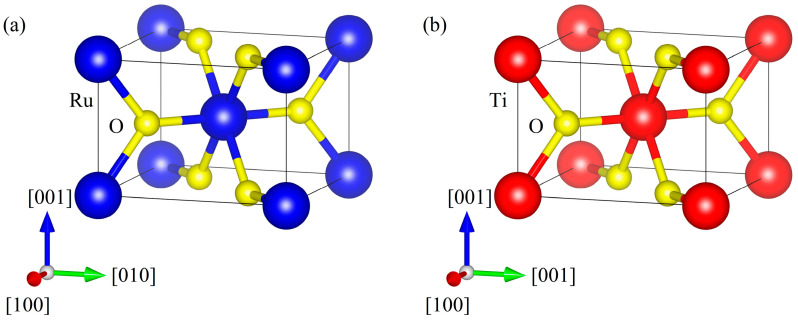
(**a**) The atomic structure of RuO_2_; (**b**) the atomic structure of TiO_2_.

**Figure 2:**
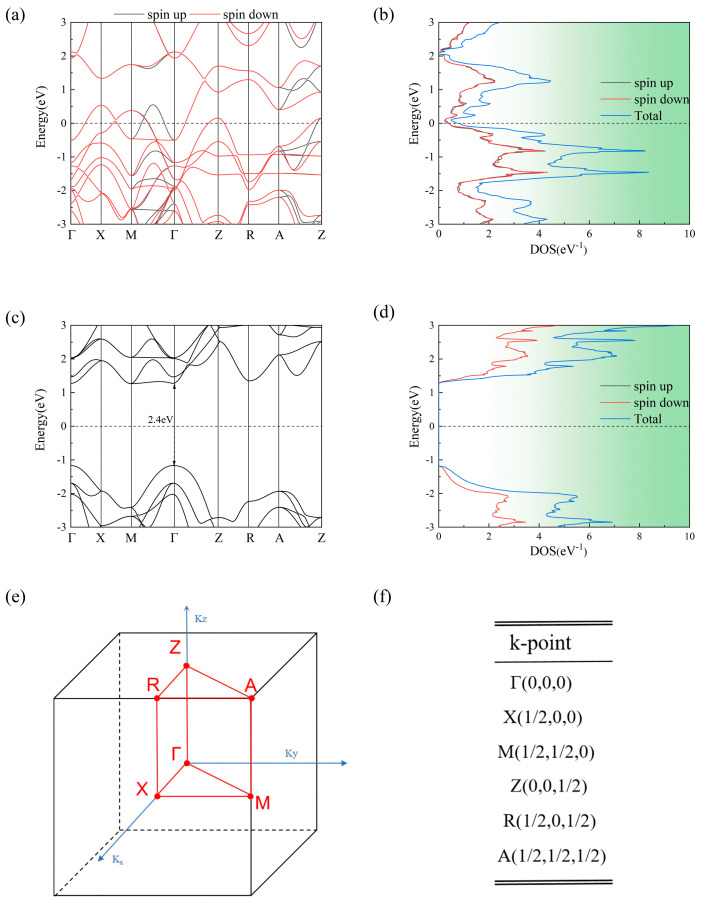
(**a**) Band structure and (**b**) DOS for RuO_2_; (**c**) band structure and (**d**) DOS for TiO_2_. (**e**) The 3D Brillouin zone of RuO_2_; (**f**) coordinates of RuO_2_’s high-symmetry points.

AFMTJ consists of two infinite RuO_2_ electrodes sandwiching rutile structure TiO_2_, which exhibits a lattice mismatch of 1.7% relative to RuO_2_. Such a small lattice mismatch rate makes the structure of AFMTJ stable and conducive to optimization. The electronic and magnetic properties of all these structures are consistent with previous results [35], indicating that our current calculations are reliable. First, we relaxed the lattice constant. Figure 1 presents the optimized structural configurations of RuO_2_ and TiO_2_. The spin-dependent band structure and density of states (DOS) of RuO_2_ and TiO_2_ are shown in Figure 2. As shown in Figure 2a, RuO_2_ exhibits metallic characteristics. The spin degeneracy along the Г-X, Г-Z, X-M, Z-R, and R-A symmetry directions is resolved in the RuO_2_ band structure, while significant spin splitting emerges along the Г-M and Z-A directions.

RuO_2_ exhibits metallic characteristics with both conduction and valence bands crossing the Fermi level. In contrast, TiO_2_ possesses a substantial direct band gap of 2.4 eV, where both bands are positioned away from the Fermi level [46].

**Figure 3:**
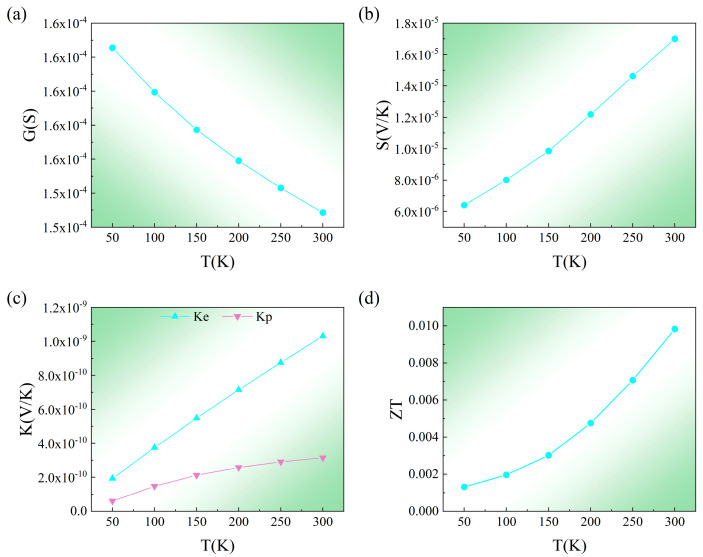
The calculated plots of (**a**) electrical conductivity, (**b**) Seebeck coefficient, (**c**) electronic and phonon thermal conductivity, and (**d**) ZT value versus temperature for RuO_2_.

The expected thermoelectric parameters and ZT value for RuO_2_ are shown in Figure 3. In contrast to the declining electrical conductivity, which is suppressed by electron–phonon scattering, the thermal contributions from electrons and phonons grow as temperature increases. Notably, the room-temperature ZT reaches 0.01, suggesting RuO_2_’s potential as a promising thermoelectric material. The ZT value of RuO_2_ (0.01 at 300 K) is higher than that of the prototypical altermagnet MnTe_2_ (ZT = 0.008 at 300 K) [47], and its momentum-locked spin splitting enables superior spin-polarized thermal transport critical for device integration. This trade-off between thermoelectric efficiency and spin functionality highlights the material-specific optimization pathways within the altermagnetic family.

**Figure 4:**
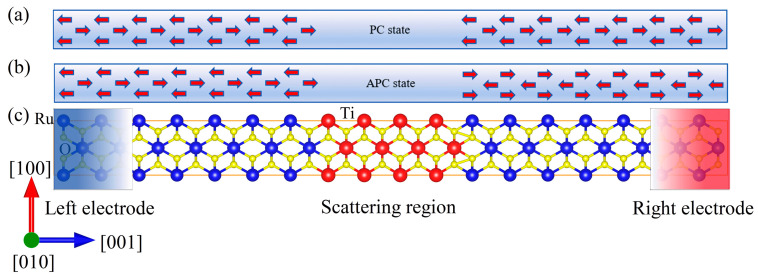
RuO_2_/TiO_2_/RuO_2_ device: (**a**) magnetic moment arrangement of PC state; (**b**) magnetic moment arrangement of APC state. (**c**) Schematic diagram of the RuO_2_/TiO_2_/RuO_2_ device. The red arrow indicates the direction of the magnetic moment on the Ru atom.

Next, we designed an AFMTJ using RuO_2_ (001) as electrodes and TiO_2_ (001) as an insulating barrier layer. Due to both RuO_2_ and TiO_2_ having a rutile structure and a similar lattice constant, this AFMTJ is experimentally feasible, as evidenced by the successful epitaxial growth of RuO_2_/TiO_2_ heterostructures via magnetron sputtering on TiO_2_ (001)-oriented substrates [48]. Figure 4c shows the atomic structure of the RuO_2_/TiO_2_/RuO_2_ (001) AFMTJ. The scattering region includes 8 TiO_2_ layers in the center and 10 RuO_2_ layers on each side, two infinitely extended RuO_2_ layers as electrodes. The rutile phase was selected for both RuO_2_ and TiO_2_ due to the following reasons: For RuO_2_, the rutile phase is its sole stable polymorph. In the case of TiO_2_, the rutile phase exhibits superior thermodynamic stability compared to other polymorphs. Crucially, when both oxides adopt the isostructural rutile configuration, their lattice mismatch is minimized, rendering this system particularly suitable for tunnel junction applications [49]. Parallel spin configuration (PC) and antiparallel spin configuration (APC) of the Ru atoms on the left and right electrodes are shown on top of Figure 4a,b.

**Figure 5:**
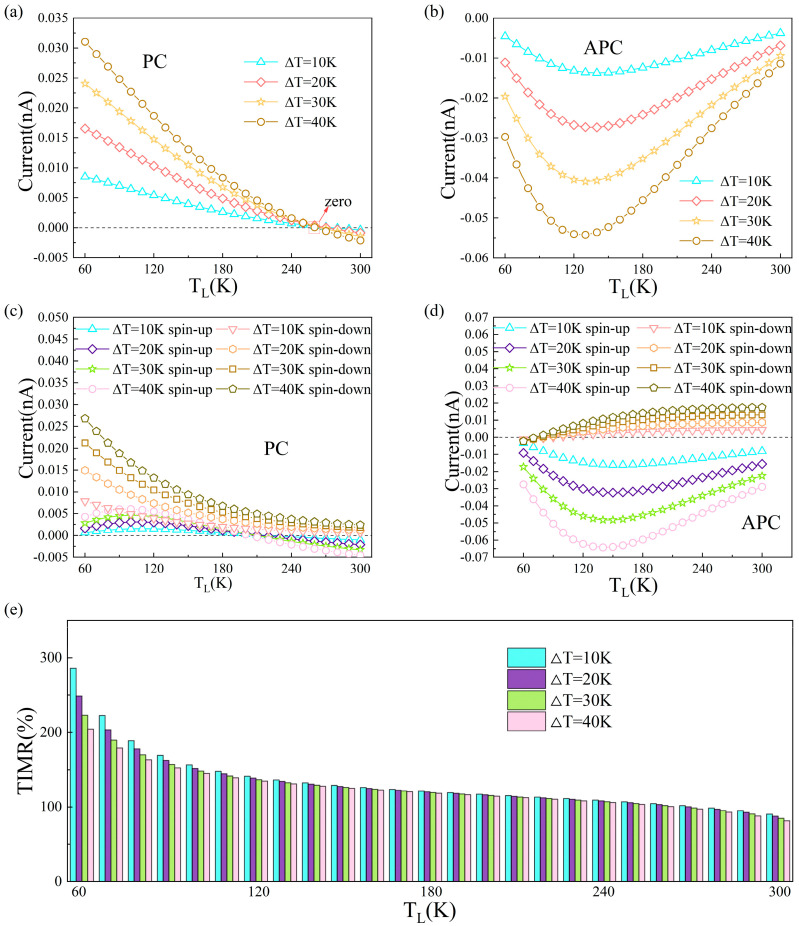
Plot of thermal spin polarization current versus T_L_ for RuO_2_/TiO_2_/RuO_2_ device in the (**a**) PC and (**b**) APC; (**e**) TIMR versus T_L_. The T_L_ dependence of thermal spin total current for the RuO_2_/TiO_2_/RuO_2_ device is shown in the (**c**) PC and (**d**) APC.

**Figure 6:**
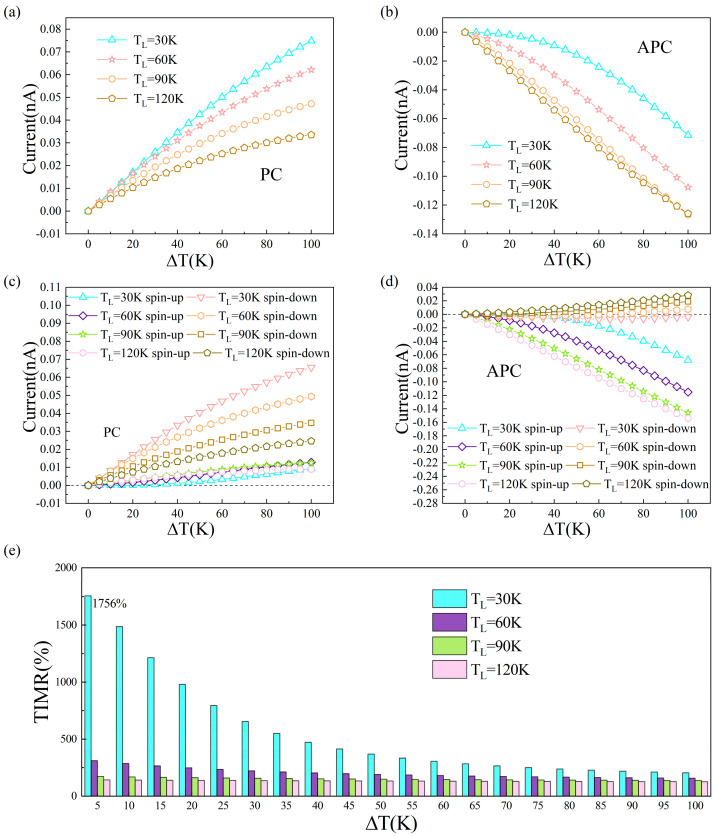
Plot of thermal spin polarization current versus ΔT for RuO_2_/TiO_2_/RuO_2_ device in the (**a**) PC and (**b**) APC; (**e**) TIMR versus ΔT. Plot of thermal spin total current versus ΔT for RuO_2_/TiO_2_/RuO_2_ device in the (**c**) PC and (**d**) APC.

The operational temperature range in this study is intrinsically limited by the Néel temperature of RuO_2_ (T_N_ ≈ 400 K) [50]. Above the T_N_, RuO_2_ undergoes a magnetic phase transition from antiferromagnetic to paramagnetic states, accompanied by electronic structure modifications that eliminate spin polarization. This invalidates the premise of our model. Concurrently, the temperature dependence of TIMR becomes negligible at room temperature. Therefore, considering T_N_ and this diminished temperature dependence, we restrict our calculations to the range of approximately 0 K to 400 K, without extending to higher temperatures. The current caused by the temperature difference (ΔT = T_R_ − T_L_) between the temperature of the left electrode (T_L_) and the temperature of the right electrode (T_R_) is plotted in Figure 5. As shown in Figure 5a,c, the total current, the spin-up current, and the spin-down current versus T_L_ at ΔT = 10 K, 20 K, 30 K, and 40 K for PC. It can be observed that a marked disparity emerges between the spin-up and the spin-down current, and the spin-down currents consistently exceed their spin-up currents with TL increasing to 260 K for different ΔT. The total current equals zero when T_L_ reaches 260 K, where spin-up and spin-down electrons flow in different directions, contributing to a net spin current. For APC, the spin-up currents consistently exceed their spin-down currents with T_L_ increasing for different ΔT, and there is no net spin current for different T_L_. The difference in the magnitude of the spin-polarized current between the PC and APC states correlates well with the difference in the magnitude of the transmission coefficient shown in Figure S3. Based on thermal current characteristics, the TIMR was derived as shown in Figure 5e, under different ΔT, TIMR values decrease with increasing T_L_. It is also very interesting to study the ΔT dependence of total current and spin current with T_L_ fixed, shown in Figure 6. Here, we fix the left electrode at 30, 60, 90, and 120 K and set the ΔT at 0–100 K. Throughout the entire ΔT range, for PC, spin-down currents remain larger than their spin-up counterparts. For APC, spin-up currents play a dominant role in the total current. As shown in Figure 6e, TIMR values under different T_L_ exhibit a platform with increasing ΔT. And when T_L_ = 30 K, the maximum TIMR can reach 1756%. The spin polarization of altermagnetic materials is a key parameter for TIMR. Increasing temperature reduces spin polarization [51] while simultaneously enhancing thermal movement of electrons and increasing their scattering probability, consequently lowering TIMR. Altermagnets uniquely combine the advantages of both ferromagnets and antiferromagnets, offering a promising platform for advanced spintronic devices. Compared with conventional ferromagnetic MTJs, altermagnetic MTJs exhibit significantly reduced stray fields due to their compensated antiparallel spin structure, minimizing crosstalk in high-density integration. Simultaneously, they overcome the limitation of weak spin-dependent signals typically associated with antiferromagnetic MTJs by generating strong momentum-dependent spin splitting, enabling robust spin-polarized transport comparable to ferromagnetic systems. This synergy of low stray fields and robust spin-polarized transport endows altermagnetic MTJs with unique advantages in realizing high-density, high-speed, and high-stability spintronic devices.

**Figure 7:**
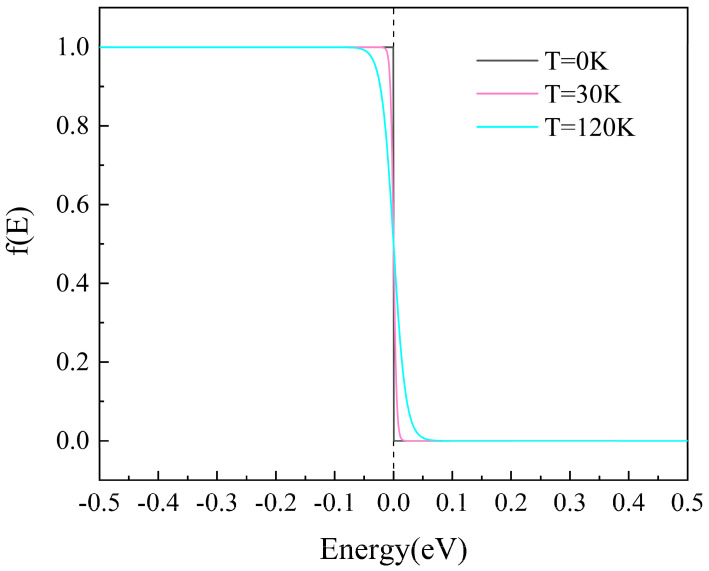
The Fermi distribution of the electrode at different temperatures.

**Figure 8:**
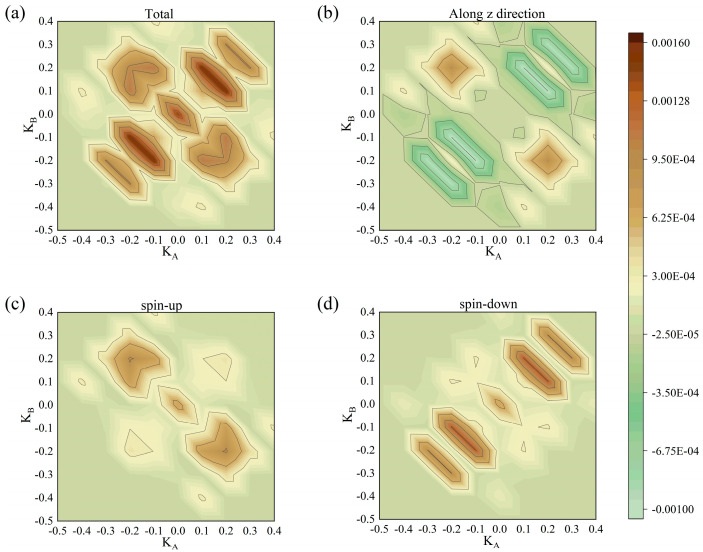
Transmission spectra of RuO_2_/TiO_2_/RuO_2_ devices in the PC at (**a**) total, (**b**) along the z-direction, (**c**) spin-up, and (**d**) spin-down.

**Figure 9:**
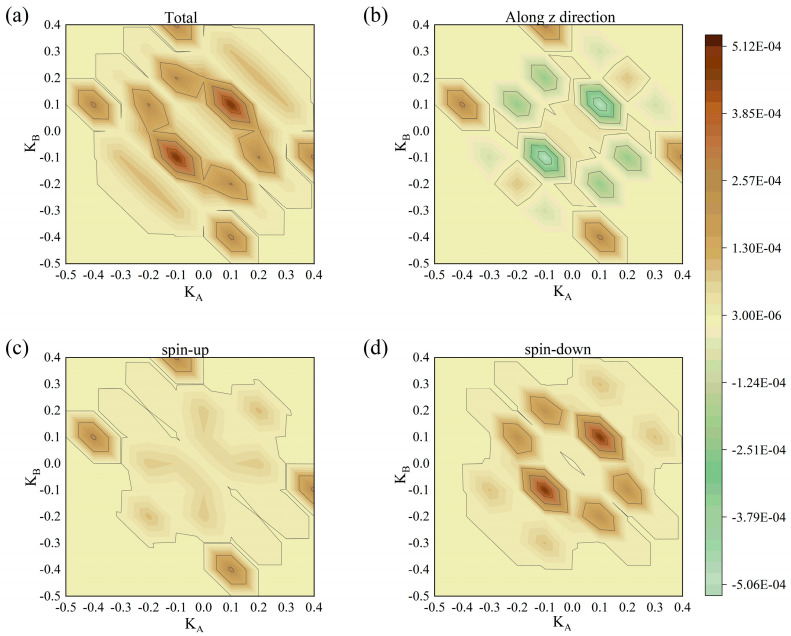
Transmission spectra of RuO_2_/TiO_2_/RuO_2_ devices in the APC at (**a**) total, (**b**) along the z-direction, (**c**) spin-up, and (**d**) spin-down.

The mechanism of this phenomenon described above is the interaction of the Fermi-Dirac distribution of the electrodes and the spin-polarized transmission spectra, as demonstrated in the Fermi-Dirac distribution figure (Figure 7). Remarkably, transmission spectra under both parallel (Figure 8) and antiparallel (Figure 9) Néel vector configurations exhibit spin-polarized transport. For PC, in comparison between spin-up and spin-down electron transmission coefficients, the spin-down electrons play the dominant role. For APC, the spin-up electrons exhibit a higher transmission probability, dominating the transport behavior.

## 4. Conclusions

In conclusion, we have studied the spin-dependent thermoelectric transport properties of variable magnetic RuO_2_ and its application in AFMTJ through first-principle calculations and quantum transport simulations. As a momentum-locked altermagnetic material, RuO_2_ exhibits zero net magnetization with nonrelativistic spin splitting in its band structure, enabling localized spin polarization critical for spin caloritronic applications. The results show that the ZT of RuO_2_ reaches 0.01 due to the influence of electron transport and the phonon scattering mechanism. Furthermore, the designed RuO_2_/TiO_2_/RuO_2_ AFMTJ demonstrates significant TIMR, reaching a maximum TIMR of 1756% at a temperature gradient of 5 K. In parallel structures, the spin-down current dominates the transmission spectrum, whereas in antiparallel structures, the spin-up current governs the transmission spectrum, underscoring the spin-polarized thermal transport. The small lattice mismatch (1.7%) between RuO_2_ and TiO_2_ ensures structural stability, while spin-polarized thermal transport highlights the interplay between altermagnetism and thermoelectric efficiency. Looking forward, the high TIMR efficiency of RuO_2_-based antiferromagnetic tunnel junctions (AFMTJs) offers a direct pathway for next-generation energy harvesting systems. This capability enables the conversion of ubiquitous low-grade waste heat gradients (<150 °C) into spin-polarized currents via the spin-dependent Seebeck effect. Such currents can directly drive ultra-low-power spintronic devices—including wireless sensor network nodes, biomedical implants, or non-volatile memory switching units—without intermediate power conversion stages. Crucially, RuO_2_’s room-temperature functionality and compatibility with CMOS processing could accelerate the development of self-powered, energy-autonomous electronics, leveraging waste thermal energy from industrial processes or microelectronic systems. These findings underscore the potential of altermagnetic materials in bridging the gap between antiferromagnetic robustness and ferromagnetic spin functionality, advancing high-efficiency, low-energy spintronic devices for waste heat recovery and spin-based memory technologies.

## Data Availability

The data presented in this study are available upon request from the corresponding authors.

## Supplementary Materials

The following supporting information can be downloaded at: https://www.mdpi.com/article/10.3390/nano15141129/s1, Figure S1. Spin-resolved HOMO and LUMO of RuO_2_/TiO_2_/RuO_2_ device under PC state. Figure S2. Spin-resolved HOMO and LUMO of RuO_2_/TiO_2_/RuO_2_ device under APC state. Figure S3. Transmission coefficient versus energy of RuO_2_/TiO_2_/RuO_2_ devices at (a) total, (b) along the z-direction, (c) spin-up and (d) spin-down.
