# Supplementary material for: Investigation of Thermoelectric Properties in Altermagnet RuO2
Source: Nanomaterials (Basel). 2025 Jul 21;15(14):1129. doi: 10.3390/nano15141129 (PMC12300824; doi:10.3390/nano15141129)
Supplement: Supplementary file 1 [file nanomaterials-15-01129-s001.zip › nanomaterials-3704066-supplementary.pdf]

Supporting Information

Investigation of Thermoelectric Properties in Altermagnet RuO_2

Jun Liu, Chunmin Ning , Xiao Liu , Sicong Zhu *and Shuling Wang *

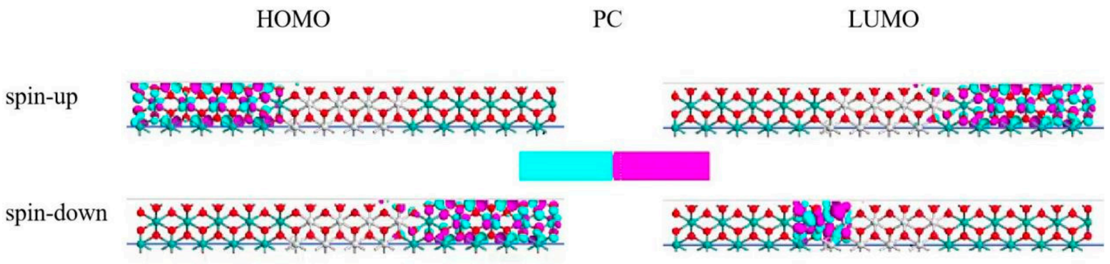

Figure S1. Spin-resolved HOMO and LUMO of $RuO_2/TiO_2/RuO_2$ device under PC state.

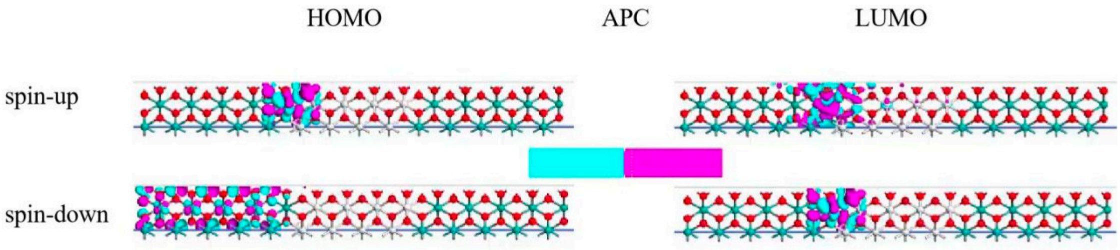

Figure S2. Spin-resolved HOMO and LUMO of $RuO_2/TiO_2/RuO_2$ device under APC state.

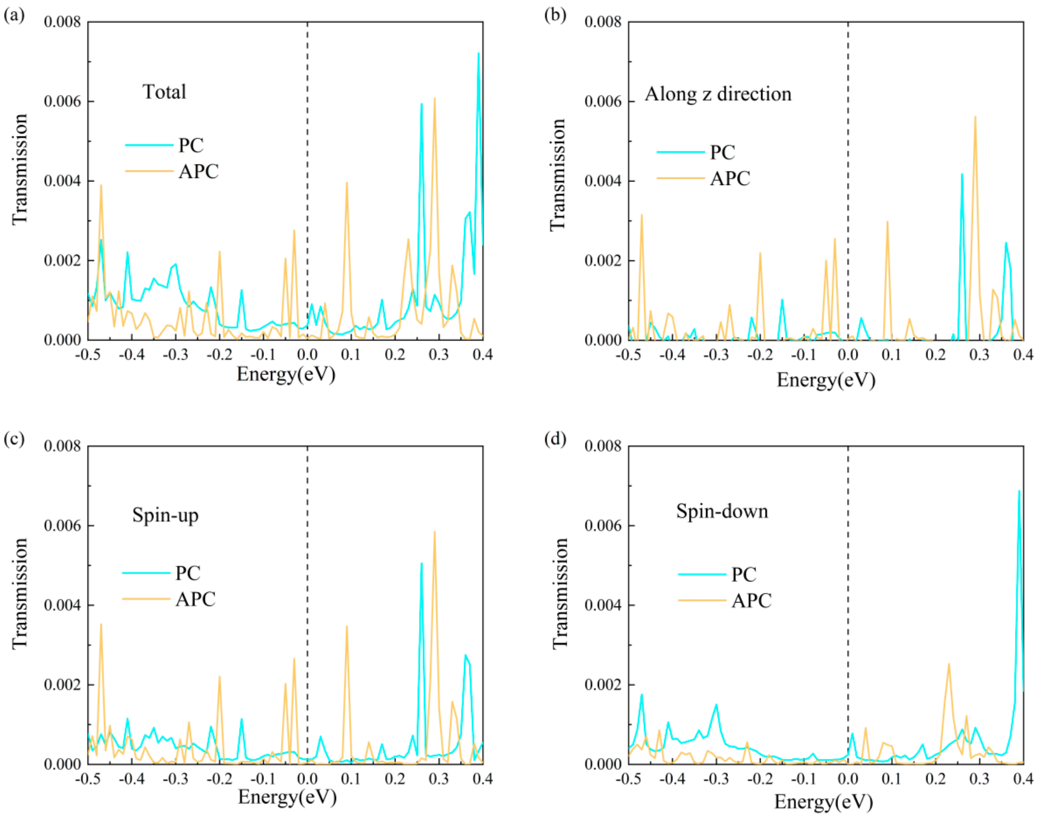

Figure S3. Transmission coefficient versus energy of $RuO_2/TiO_2/RuO_2$ devices at (a) total, (b) along the z-direction, (c) spin-up and (d) spin-down.
